# Knifefish’s suction makes water boil

**DOI:** 10.1038/s41598-020-75788-x

**Published:** 2020-10-29

**Authors:** Victor M. Ortega-Jimenez, Christopher P. J. Sanford

**Affiliations:** Department of Ecology, Evolution, and Organismal Biology, Kennesaw State University, Kennesaw, GA 30144 USA

**Keywords:** Biomechanics, Fluid dynamics

## Abstract

We discovered that knifefish (*Apteronotus albifrons*) during suction feeding can produce millimeter-sized cavitation bubbles and flow accelerations up to ~ 450 times the acceleration of gravity. Knifefish may use this powerful suction-induced cavitation to cause physical damage on prey hiding in narrow refuges, therefore facilitating capture.

## Introduction

Cavitation bubbles in a liquid are induced by intense and sudden changes of local pressure^1^. These collapsing bubbles can produce intense compressional waves and extremely high temperatures, as well as serious damage on structures. In nature, besides mantis^2^ and pistol shrimps^3^, there is no other taxon known that uses cavitation for prey capture. This is particularly intriguing because feeding in several aquatic organisms^4,5^, such as fishes, rely on generating rapid low pressures inside the buccal cavity^6,7^.

**Figure 1:**
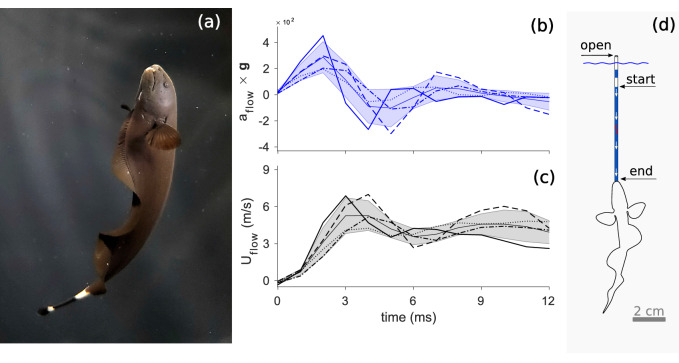
(**a**) Black ghost Knifefish (*Apteronotus albifrons*). (**b**) Flow acceleration (blue) and (**c**) speed (black) over time produced by suction in a capillary tube open to the air. Lines represent time series of each sampled individual. Starting time is when acceleration increases from zero. Shadows represent average value and one SD (N = 4). (**d**) Drawing represents the experimental setup. This figure was created using MATLAB R2017b (https://www.mathworks.com/), GIMP 2.8 (free available at: https://www.gimp.org/), and Inkscape 0.92.3 (free available at: https://inkscape.org/).

Knifefish are nocturnal freshwater fishes that thrive in Central and South American streams and rivers. They are well known for their remarkable electrical senses, slender morphology, and anal fin propulsion^8,9^ (Fig. 1a). In the wild, knifefish feed mostly on aquatic invertebrates, such as insects^10^, which can perform rapid evasive maneuvers. Furthermore, prey can hide inside tiny holes of submerged logs, between rocks or in the mud, making it difficult for a predator to reach, detach and extract them.

## Results

We discovered that knifefish while generating suction at the tip of an 8 mm long capillary tube (1.5 mm diameter), open to the air, induces a rapid jet of water toward the mouth which in turn can generate cavitation bubbles (Video S1). Approximately 3 ms after the onset of suction, we registered a maximum flow speed and acceleration (a_max_) of up to ~ 7 m/s and ~ 4500 m/s^2^ respectively (Fig. 1). In the open tube experiments we confirmed cavitation bubbles appearing close to the mouth in three individuals and four of the 16 sampled trials. It is noteworthy that in all trials we observed marked flow instabilities appearing in the air–water interface inside the open tube during suction (Video S1), which may be related to cavitation.

**Figure 2:**
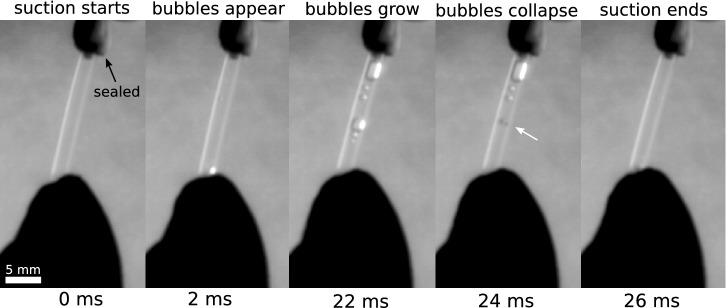
Frame sequence filmed at 500 frames/s of a fish suction feeding a the tip of a sealed tube, showing large cavitation bubbles. This figure was created using GIMP 2.8 (free available at: https://www.gimp.org/) and Inkscape 0.92 (free available at: https://inkscape.org/).

It has recently been demonstrated that cavitation induced by an impulsive system can be better defined by acceleration rather than speed^1^. Here we calculated the cavitation number as Ca = (p_r_ − p_v_)/(ρLa_max_)^1^, where p_r_ is the atmospheric pressure, p_v_ is the vapor pressure, ρ is the water density, and L is the distance between the start and end positions of water level in the tube, respectively. We found that knifefish suction feeding generates Ca of 0.65 (± 0.20 SD, N = 4), which sufficiently meets the criteria to generate cavitation (Ca < 1). We excluded the extra pressure required to overcome resistance in the capillary tubes (i.e., pressure drop), which could result in even lower cavitation numbers. Remarkably, when fish suction feed from a tube sealed at one end, several cavitation bubbles appeared, grew, and then collapsed during a 22 ms period (Fig. 2). All four individuals produced cavitation bubbles in the sealed tube. A hammer-like sound during suction was also heard (Video S1) that seemed to coincide with collapsing of the bubble (Video S2).

**Figure 3:**
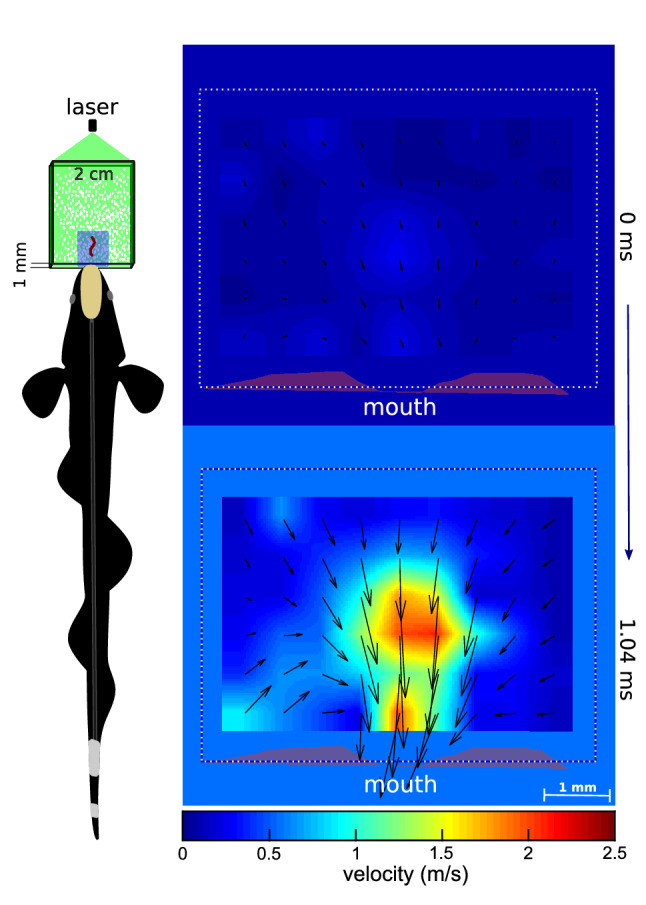
Flow velocity fields using PIV produced by a fish generating suction between two transparent plates, at the beginning (top) and after ~ 1 ms (bottom). Drawing represents the experimental setup. For details see Supplementary information and video S1. This figure was created using MATLAB R2017b (https://www.mathworks.com/) and Inkscape 0.92.3 (free available at: https://inkscape.org/).

To investigate the fluid dynamics produced by the knifefish suction feeding between two parallel plates (1 mm separation) we performed 2D particle image velocimetry, filming at ~ 7000 frames/s. We found that a knifefish generates very close-range suction (~ 3 mm) in front of the mouth. Flow speed in this region increased from ~ zero to ~ 2.5 m/s in one millisecond (i.e., an acceleration of ~ 2500 m/s^2^, Fig. 3).

## Discussion

This study demonstrates that black ghost knifefish can produce extreme suction resulting in a powerful jet, a hammer-like sound, and cavitation bubbles in a capillary tube. Therefore, we suggest that this nocturnal and riverine fish may use cavitation to facilitate prey extraction, capture and intake of small prey, hiding inside the matrix of submerged vegetation or between microcracks of rocks. We observed that knifefish were able to extract the air inside a bamboo stick by producing suction at the tip (Video S2 [00:10 s]). It is known that snapping shrimp can also use a powerful jet, followed by cavitation bubbles to stun and kill prey^3^. Thus, knifefish can generate cavitation using a different mechanical system from invertebrates. This finding represents the first example of cavitation we are aware of in vertebrate systems.

Furthermore, cavitation may facilitate prey immobilization, dislodgement and capture. Invertebrates, such as copepods, exposed to ultrasonic cavitation can experience damage and can even break apart^11^. Another possibility is that sound waves produced during bubble collapse (Video S2) may be useful to pinpoint and evaluate prey located in confined spaces. Vapor bubbles can be detected using a rebound shock wave during collapse^12^. This has the potential to be a useful mechanism of prey detection as electroreception in knifefishes can be impaired by cluttered environments^13^.

It is important to consider the negative effects produced by cavitation on those organisms that generate it. Mantis shrimps, for example, produce cavitation via a powerful strike, which can damage their own appendages^2^. However, their appendages are well designed to resist such impact forces^14^. Thus, we can expect that cavitation bubbles produced in the oral cavity of knifefish could also cause certain damage. Future work on knifefishes in their natural habitat will reveal important clues regarding the role of cavitation in their ecology.

Particle image velocimetry demonstrates that knifefish suction-feeding can generate flow speeds of 2.5 m/s in ~ 1 ms, and an average flow acceleration of 2500 m/s^2^. This aligns well with the measurements of knifefish when generating suction on an open capillary tube (Fig. 1). Our results suggest that higher flow speeds and lower pressures are likely generated inside the mouth of a knifefish. Seahorses for example, can reach flow speeds inside the mouth cavity up to 3.5 m/s^15^. When compared to other fishes, knifefish generate suction flow speeds that are two to three times higher than those generated by sunfish (max 0.8 m/s in 2 ms^16^) and largemouth bass (1.4 m/s in 4 ms^17^), respectively. However, in comparison to pistol shrimps, flow speed and acceleration in knifefish are modest. A computational model indicates that pistol shrimp can generate a jet of water with a peak speed up to 30 m/s in 0.3 ms^18^, which corresponds to an acceleration of 10^5^ m/s^2^. Nevertheless, knifefish are still capable of producing cavitation at lower speeds.

In conclusion, we found that knifefish under laboratory conditions can produce cavitation bubbles during suction feeding. To our knowledge this is the first reported example of induced cavitation in a vertebrate system. It is possible that this capability may serve an important role for immobilization, dislodgement, and capture of prey. Furthermore, it could be used as a method of prey detection in narrow refuges. Future comparative work among suction feeding organisms, such as carnivorous plants, amphibians and other fishes will establish if this ability to cavitate is more widespread than has been previously thought.

## Methods

### Ethics statement

All methods were carried out in accordance with relevant federal guidelines and regulations. All training and experimental procedures were approved by Kennesaw State University’s Institutional Animal Care and Use Committee (ACUP #20-005), and no animals were sacrificed for this study.

### Fish suction on an open tube

Four black ghost knifefish (*Apteronotus albifrons*) were sampled. Fish were acquired from a local aquatic pet store at Kennesaw, Georgia. Each individual was housed in a 151 L tank with a PH of 7–8 and a water temperature of 25–26 °C. Animals were fed frozen blood worms *ad-libitum*. During experiments, each individual was transferred to a small experimental tank (19 L). They were trained for two days to feed on blood worms introduced to a plastic clear tube (internal diameter 1.5 mm, external diameter 2.5 mm and 8 cm long) (Fig. 1b).

One high speed camera (Fastec HiSpec 1) was used for recording at 1000 frames/s each fish feeding at the capillary tube. The tube was filled with water and introduced vertically by hand into the tank. The top end of the tube was open to the air. Air pockets inside the tube were used to track the distance traveled during suction. A sheet of white paper placed outside the tank was used to provide contrast. Two LED lights illuminated the capillary tube. In this study, because we were interested in maximal performance of knifefish, only one sequence for each individual was analyzed in which flow acceleration was maximal. Variation between each sampled fish (4 trials per fish) with body size are shown in Figure S1.

For each sequence (one for each fish) we digitized the vertical position of the water level (i.e., the air pocket interface) in the tube using the DLTdv5.m digitizing tool for Matlab (https://biomech.web.unc.edu/dltdv/). Flow speed and acceleration induced by suction were calculated from the first and second derivatives of the MSE-quintic spline function^19^.

### Cavitation number

We calculated the cavitation number as follows Ca = (p_r_ − p_v_)/(ρLa_max_), where p_r_ is the atmospheric pressure (100 kPa), p_v_ is the vapor pressure at 25 ºC (3.17 kPa), ρ is the water density (1 × 10^3^ kg/m^3^), L is the distance between the start and end positions of water level in the tube (6 ± 1 cm), and a_max_ is the maximal flow acceleration (2800 ± 1200 m/s^2^), respectively. We found that Ca was 0.65 ± 0.20. Data is presented as average value ± one SD (N = 4).

### Suction on sealed tube

We performed similar experiments to those described above. However, in this case the fish was allowed to suction feed at the tip of a capillary tube (internal diameter 1.5 mm, external diameter 2.5 mm and 4 cm long) filled with water, and hermetically sealed on one end (Fig. 1c). Similarly as above, we filmed each fish while suction feeding at the tip of the tube at 500 frames/s.

### Particle image velocimetry

A class-4 laser (Opto Engine LLC, 532 nm, 5 W) was used to produce a horizontal light sheet to illuminate plastic beads (~ 50 µm) introduced between two parallel plastic plates. Each plate was 2 × 3 cm with a thickness of 0.5 mm. Separation between plates was 1 mm. A fish was trained to suction feed on blood worms placed between both plates (Fig. 1d). We filmed the top view using a high-speed camera (Fastec HiSpec 4) at 6754 frames/s and used paired frames from the recorded video sequence to calculate velocity fields using PIVlab (https://pivlab.blogspot.com/2017/07/pivlab-direct-download.html)^20^. A ROI of 163 × 121 pixels was sampled. An interrogation window from 64 × 64 pixels to 32 × 32 pixels, excluding those vectors with standard deviation greater than 5, was used.

## Supplementary information

Supplementary Information 1.

Supplementary Information 2.

Supplementary Video 1.

Supplementary Video 2.

## Data Availability

The datasets supporting this article have been uploaded as part of the electronic supplementary material.

## Code availability

The PIV and Digitization software that support the findings of this study are available in https://pivlab.blogspot.com/2017/07/pivlab-direct-download.html and https://biomech.web.unc.edu/dltdv/ respectively.
