# Supplementary material for: Knifefish’s suction makes water boil
Source: Sci Rep. 2020 Oct 29;10:18698. doi: 10.1038/s41598-020-75788-x (PMC7596043; doi:10.1038/s41598-020-75788-x)
Supplement: Supplementary file 2 — Supplementary Information 2. [file 41598_2020_75788_MOESM2_ESM.pdf]

SUPPLEMENTARY MATERIALS

Knifefish's Suction Makes Water Boil

Victor M. Ortega-Jimenez[1], Christopher P. Sanford[1]

[1]Department of Ecology, Evolution, and Organismal Biology. Kennesaw State University, Kennesaw, GA

Corresponding author: ornithopterus@gmail.com

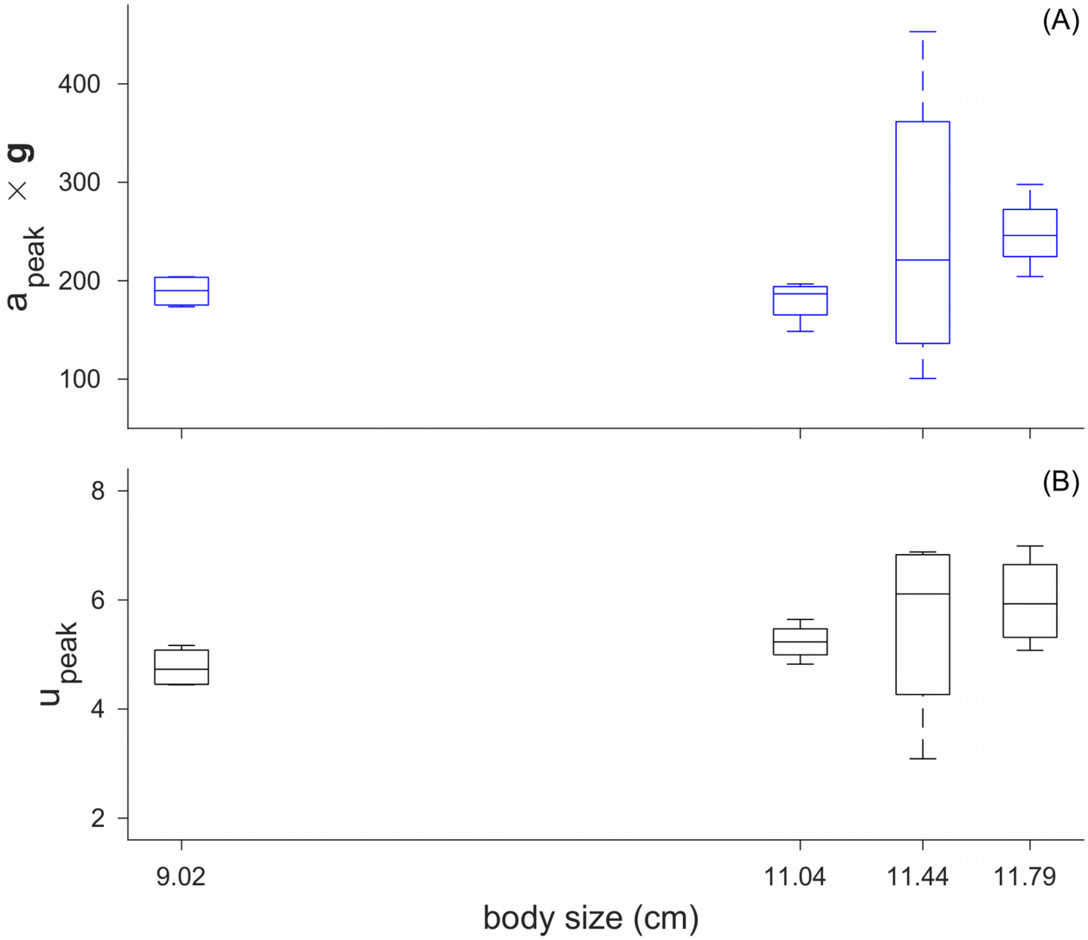

Figure S1. Peak flow acceleration (a) and speed (b) versus body size. Boxplots (n=4 for each fish) represent medians and 25-75 percentiles.

VideoS1.mp4 [(00:21 s) Knifefish producing a cavitation bubble during suction on a capillary tube open to the air. (00:33 s) Knifefish producing several cavitation bubbles during suction on a sealed tube. (00:38 s) Knifefish accelerating flow during suction on a sealed capillary tube. (01:06 s) Flow velocity field produced by a Knifefish during suction between two transparent plates (separation 1 mm)].

VideoS2.mp4 [(00:00s) Knifefish producing a hammer-like sound and cavitation bubbles during suction feeding. (00:05s) Hammer-like sound produced during bubble collapse. (00:10s) Knifefish generating suction and extracting air from the tip of a bamboo stick. Video was recorded with a Samsung S10 phone at 240 frames/s. Sound levels were increased for clarity]

Rawdata_KnifefishSuctionAll_R1.xlsx [Raw Digitization and MSE-quintic spline data. Time (ms), Y-position (cm), spline-Y-position (cm), speed (cm/s) and acceleration (cm/s^2). Data for each fish (four individuals and four trials each) are in each sheet. Digitization software is described in: Hedrick, T. L. (2008). Software techniques for two- and three-dimensional kinematic measurements of biological and biomimetic systems. Bioinspir. Biomim. 3, 034001]. Body Mass for each fish is as follows: (Fish A: 11.79 cm; Fish B: 11.04 cm; Fish C: 11.44 cm; and Fish D: 9.02 cm).

MATLAB CODE TO READ RAW DATA :

```
clear all; close all; clc;
[~,sheet_name]=xlsfinfo('Raw_position_knifefishSuction_R1.xlsx')
for k=1:numel(sheet_name)
  data{k}=xlsread('Raw_position_knifefishSuction_R1.xlsx',sheet_name{k})

T=data{1,k}(:,1);  %time
py=data{1,k}(:,2);  %Y position cm
phaty=data{1,k}(:,3);  %Smoothed y Position cm
pdy=data{1,k}(:,4); %  %speed dy cm/s
pddy=data{1,k}(:,5);   %% acceleration ddy cm/s^2

uiy=-pdy(1:end)/100;   %speed m/s
aiy=-pddy(1:end)/(100); % acceleration m/s



u_maxY=max(uiy);  % peak speed
a_maxy=max(aiy);   %peak acceleration

g=9.81; %gravity acceleration m/s2


Nf=14; %%frames to plot


MaxA(1:length(a_maxy),k)=a_maxy;  %all fish data - peak acceleration
MaxU(1:length(u_maxY),k)=u_maxY;  %all fish data - peak speed


Mui(1:length(uiy),k)=uiy;  %speed time series all fish
Mai(1:length(aiy),k)=aiy;   %acceleration time series all fish



clearvars -except sheet_name data Mui Mai MaxA MaxU Nf
end

%%%%%%%%%%%%%%% FIGURE 1c,d

fig=figure(1)
annotation('textbox',[0.85,0.95,0.16,0],'String',{'(A)'},'FontSize',12,'Color','k','EdgeColor','none')
annotation('textbox',[0.85,0.48,0.16,0],'String',{'(B)'},'FontSize',12,'Color','k','EdgeColor','none')
annotation('textbox',[0.092,0.975,0.58,0],'String',{'\times 10^2'},'FontSize',8,'Color','k','EdgeColor','none')

T=(0:(1/1000):(Nf-1)/1000);
Mui2= [Mui(10:23,9) Mui(13:26,11) Mui(17:30,12) Mui(13:26,16)];  %plotting when speed started
Mai2= [Mai(10:23,9) Mai(13:26,11) Mai(17:30,12) Mai(13:26,16)];  %plotting when acceleration started

T=T';  % time in ms
subplot(2,1,2)
hold on
```

```
shadedErrorBar(1000*T,Mui2',{@mean,@std},{'k','markerfacecolor','k'});
ylabel('U_f_l_o_w (m/s) ','FontSize',20); %% tangential speed
xlabel('time (ms)','FontSize',20);
set(gca,'YTick',[0:3:9],'TickDir','out','FontSize',15)
set(gca,'XTick',[0:3:12],'TickDir','out','FontSize',15)
box off
  ylim([-0.5, 9.3]);
  xlim([-0.02,12]);
  %%%time series per individual
 plot(1000*T,Mui2(:,1),'k-','linewidth',1.1)
 plot(1000*T,Mui2(:,2), 'k--','linewidth',1.1)
 plot(1000*T,Mui2(:,3), 'k:','linewidth',1.1)
 plot(1000*T,Mui2(:,4),'k-.','linewidth',1.1)


subplot(2,1,1)
hold on

shadedErrorBar(1000*T,Mai2'./(100*9.81),{@mean,@std},{'b','markerfacecolor','b'});
ylabel('a_f_l_o_w \times \bf{g} ','FontSize',20);

set(gca,'YTick',[-4, -2, 0, 2, 4],'TickDir','out','FontSize',15)
set(gca,'XTick',[0:3:12],'TickDir','out','xticklabel', [],'FontSize',15)
box off
  ylim([-4.3, 5.2]);
  xlim([-0.02,12]);

%time series per individual
 plot(1000*T,Mai2(:,1)./(100*9.81),'b-','linewidth',1.1)
 plot(1000*T,Mai2(:,2)./(100*9.81),'b--','linewidth',1.1)
 plot(1000*T,Mai2(:,3)./(100*9.81),'b:','linewidth',1.1)
 plot(1000*T,Mai2(:,4)./(100*9.81),'b-.','linewidth',1.1)




%%%%%%%%%%%%%%%%%%%%%%%%%%%%%%%%%%%%%%%%%%%%%%%%%%%%%%%%

%%%%%%%%%%%%FIGURE S1


%%%Acceleration per fish
indA=[MaxA(1) MaxA(2) MaxA(10) MaxA(11)]; %Fish A
indB=[MaxA(3) MaxA(4) MaxA(5) MaxA(12)]; % Fish B
indC=[MaxA(6) MaxA(7) MaxA(8) MaxA(9)]; %Fish C
indD=[MaxA(13) MaxA(14) MaxA(15) MaxA(16)]; % Fish D

%speed per fish
indAu=[MaxU(1) MaxU(2) MaxU(10) MaxU(11)]; %fish A
indBu=[MaxU(3) MaxU(4) MaxU(5) MaxU(12)]; % Fish B
indCu=[MaxU(6) MaxU(7) MaxU(8) MaxU(9)]; %Fish C
indDu=[MaxU(13) MaxU(14) MaxU(15) MaxU(16)]; %Fish D
```

```
A_all=[indA;indB;indC;indD];
A_allU=[indAu;indBu;indCu;indDu];
A_allg=A_all./9.81;

size=[11.79 11.04 11.44 9.02];%Fish Size in cm
ni=[1:16];

figure(2)
annotation('textbox',[0.85,0.95,0.16,0],'String',{'(A)'},'FontSize',11,'Color','k','EdgeColor','none')
annotation('textbox',[0.85,0.48,0.16,0],'String',{'(B)'},'FontSize',11,'Color','k','EdgeColor','none')

subplot(2,1,1)
boxplot(A_allg', 'positions', size', 'labels',size')

ylabel('a_{peak} \times \bf{g} ','FontSize',14);
% xlabel('size (cm)','FontSize',15);
box off
a = get(get(gca,'children'),'children');   % Get the handles of all the objects
tb = get(a,'tag');   % List the names of all the objects
box1 = a(:);   % The 7th object is the first box
set(box1, 'Color', 'b');   % Set the color of the first box to green
set(gca,'YTick',[100:100:500])
set(a,{'linew'},{1})
ylim([50, 480]);

figure(2)
subplot(2,1,2)
boxplot(A_allU', 'positions', size', 'labels', size')
ylabel('u_{peak} ','FontSize',14);
xlabel('body size (cm)','FontSize',13);
box off
a1 = get(get(gca,'children'),'children');   % Get the handles of all the objects
ta = get(a1,'tag');   % List the names of all the objects
box1 = a1(:);   % The 7th object is the first box
set(box1, 'Color', 'k');   % Set the color of the first box to green
set(gca,'YTick',[2:2:8])
set(a1,{'linew'},{1})
ylim([1.6, 8.4]);
```
